# Quantitation of Glucocorticoid Receptor DNA-Binding Dynamics by Single-Molecule Microscopy and FRAP

**DOI:** 10.1371/journal.pone.0090532

**Published:** 2014-03-14

**Authors:** Femke L. Groeneweg, Martin E. van Royen, Susanne Fenz, Veer I. P. Keizer, Bart Geverts, Jurrien Prins, E. Ron de Kloet, Adriaan B. Houtsmuller, Thomas S. Schmidt, Marcel J. M. Schaaf

**Affiliations:** 1 Department of Medical Pharmacology, Leiden University/LUMC, Leiden, The Netherlands; 2 Department of Pathology, Erasmus MC, Rotterdam, The Netherlands; 3 Physics of Life Processes, Institute of Physics (LION), Leiden University, Leiden, The Netherlands; 4 Cell & Developmental Biology, Biocenter, Würzburg University, Würzburg, Germany; 5 Molecular Cell Biology, Institute of Biology, Leiden University, Leiden, The Netherlands; University of Ulm, Germany

## Abstract

Recent advances in live cell imaging have provided a wealth of data on the dynamics of transcription factors. However, a consistent quantitative description of these dynamics, explaining how transcription factors find their target sequences in the vast amount of DNA inside the nucleus, is still lacking. In the present study, we have combined two quantitative imaging methods, single-molecule microscopy and fluorescence recovery after photobleaching, to determine the mobility pattern of the glucocorticoid receptor (GR) and the mineralocorticoid receptor (MR), two ligand-activated transcription factors. For dexamethasone-activated GR, both techniques showed that approximately half of the population is freely diffusing, while the remaining population is bound to DNA. Of this DNA-bound population about half the GRs appeared to be bound for short periods of time (∼0.7 s) and the other half for longer time periods (∼2.3 s). A similar pattern of mobility was seen for the MR activated by aldosterone. Inactive receptors (mutant or antagonist-bound receptors) show a decreased DNA binding frequency and duration, but also a higher mobility for the diffusing population. Likely, very brief (≤1 ms) interactions with DNA induced by the agonists underlie this difference in diffusion behavior. Surprisingly, different agonists also induce different mobilities of both receptors, presumably due to differences in ligand-induced conformational changes and receptor complex formation. In summary, our data provide a consistent quantitative model of the dynamics of GR and MR, indicating three types of interactions with DNA, which fit into a model in which frequent low-affinity DNA binding facilitates the search for high-affinity target sequences.

## Introduction

In the past decade, imaging studies of fluorescently tagged proteins inside living cells have enormously increased our understanding of transcription factor dynamics [1], [2], [3], [4], [5], [6], [7], [8], [9]. These studies have shown that transcription factors display a remarkably high mobility in the nucleus. Even in its most activated state a typical transcription factor appears to be able to diffuse through the entire nucleus, and to be immobilized only transiently [7], [10], [11]. One often-studied transcription factor is the glucocorticoid receptor (GR). This cytoplasmically localized receptor translocates to the nucleus upon binding of naturally occurring glucocorticoids (corticosterone and cortisol) or their synthetic analogs. In the nucleus the steroid-GR complexes can bind either directly or indirectly (through interactions with other transcription factors) to DNA and alter transcription rates of responsive genes [12], [13], [14]. Like other transcription factors, ligand-activated GRs display a high mobility within the nucleus in fluorescence recovery after photobleaching (FRAP) studies [3], [4], [8], [15], [16]. Using GR mutants with reduced DNA-binding capacity or antagonist-bound GR, a correlation was shown between GR immobilization time and the capacity to initiate transcription [3], [8], [17].

In the last decade many new imaging techniques have become available that open possibilities for more detailed quantifications of protein dynamics [18], [19], [20], [21], [22]. One such approach is single-molecule microscopy (SMM). In SMM, conventional wide-field fluorescent microscopy is combined with a fast, ultra-sensitive CCD camera to enable the visualization of single fluorescent molecules with high temporal (∼5 ms) and spatial (positional accuracy of ∼40 nm) resolution [21], [23]. Initially, SMM was used to study the mobility patterns of membrane proteins [24], [25], [26], [27], [28], and it has now been adapted for studies of nuclear proteins [29], [30] and transcription factors [19], [22], [31], [32], including a recent study on the GR [18]. Importantly, the analysis of single-molecule displacement patterns gives a very direct and unbiased picture of protein dynamics [33], [34]. For the more conventional population-based approaches, the correct control for confounding factors such as laser irregularities and the requirement of many *a priori* assumptions and independent variables introduce bias in the outcomes and have been a major challenge for the field [6], [9], [15], [35]. To control for any confounding factors that might still exist in the SMM analysis, we combine SMM analysis with an established Monte Carlo quantification approach of FRAP imaging [6], [36]. The combination with FRAP not only gives independent cross-validation of the SMM predictions, but also enables a quantification of protein kinetics over a longer time frame than SMM.

Our data show that this combination of techniques provides a very consistent quantitative analysis of GR dynamics. Based on our data, we can distinguish three states of agonist-activated GR molecules; one diffusing state and two DNA-bound states, one with short (<1 sec) and one with a longer (2-4 sec) binding duration. Transcriptionally inactive GR variants show a reduction in the frequency and in the duration of both DNA binding events, and an increase in the diffusion rate of the diffusing population. This suggests that within this diffusing population an additional very brief DNA-binding event is hidden, resulting in a lower effective diffusion rate. Finally, similar effects are observed for a different steroid receptor: the mineralocorticoid receptor (MR), indicating that these data are representative for steroid receptors in general.

## Materials and Methods

### Cell line and plasmids

In most experiments, COS-1 cells were used, transiently transfected using the TransIT-COS kit (Mirus), according to the manufacturer's instructions (500 ng DNA/10 cm^2^). Transfected cells were used in experiments 2–5 days after transfection. For one experiment, Hep3B cells were used, stably transfected with the pEYFP-hGR expression vector [3]. The generation of the pEYFP-GR plasmid, the three deletion mutants of this vector (pEYFP-GR Δ9-385, pEYFP-GR Δ428-490, and pEYFP-GR Δ551-777, and the point mutant (pEYFP-GR F623A) has been described previously [3], [4]. The plasmid pEYFP-hMR was generated by PCR amplification (Phusion HF polymerase, Finnzymes) of the human MR gene from a pRSV human MR template (kindly provided by Dr. R. Evans (gene expression laboratory and HHMI, The Salk Institute for Biological Studies, La Jolla, CA).

### Single molecule microscopy

**Figure 1:**
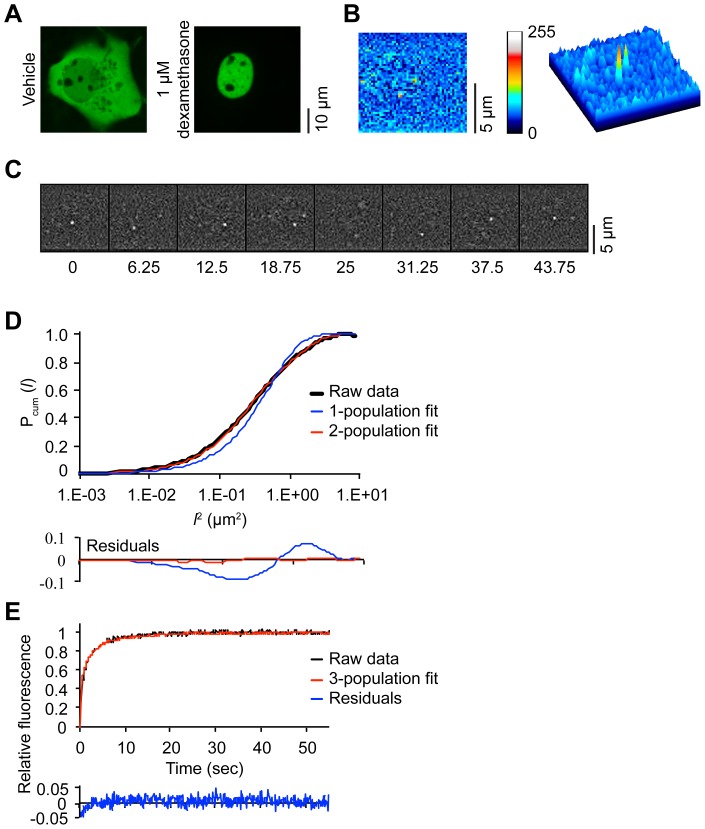
SMM and FRAP procedures. (A) Representative confocal images show complete nuclear translocation of YFP-GR after 3 hours of 1 µM dexamethasone treatment. (B) A representative CCD image of single molecules of YFP-GR after background subtraction shows two discernible Gaussian peaks of YFP fluorescence. (C) Regime for single molecule kinetics; images are taken with a time lag of 6.25 ms or 12.5 ms in 300 series of 8 per cell. In background-subtracted images, single molecules of YFP fluorescence are easily discernible. (D). PICS analysis of single molecule displacements, shown for dexamethasone-bound YFP-GR at time delay of 6.25 ms. The cumulative probability distribution as a function of the squared distance *l* (black line) is best fitted with a 2-population model (red dashed line), while a 1-population model gives a suboptimal fit (blue line) (n = 20 cells). (E) FRAP procedure of dexamethasone-bound YFP-GR. At t = 0 s a 100 ms bleach pulse is applied to a strip spanning the nucleus. Subsequently, FRAP recovery curves of 30 cells are recorded, combined and adjusted to baseline fluorescence (black line). Subsequently, Monte Carlo simulations are generated using a 3-population model and fitted to the combined FRAP curve. The top 10 fits are combined (red line) and show a good fit of the experimental data with small residuals (blue line).

Before SMM recordings, cells were exposed to 1 µM of corresponding hormones for 3–6 h. For SMM measurements, this medium was replaced by serum- and phenol red-free D-MEM medium, supplemented with 1 µM of the corresponding hormone. Subsequently, cells were transferred to the SMM setup and imaged for up to 90 min at 35°C. A wide-field fluorescence microscope (Axiovert 100TV, Zeiss) was used, equipped with a 100x/1.4NA oil-immersion objective (Zeiss). A region-of-interest (ROI) of 50×50 pixels (pixel size of 220 nm) was selected. The sample was illuminated by an 514 nm argon laser at an intensity of 2 kW/cm^2^ (measured at the object). The pulse length of 3 ms was controlled by an acusto-optical tunable filter (AA optoelectronics, France). The EYFP fluorescence signal was detected through a combination of filters (DCLP530, HQ570/80 (Chroma Technology, Brattleboro, VT) and OG530-3 (Schott, Mainz, Germany)), by a liquid-nitrogen cooled CCD camera (Princeton Instruments, Trenton, NJ), camera read out and AOTF timing were tightly controlled. Nuclei with a regular ellipsoidal appearance showing a moderate level of fluorescence were selected and photobleached until single fluorescence intensity peaks could be distinguished. The position of each individual molecule was fitted with the intensity profile of a 2D Gaussian model of EYFP peaks [37]. Our peaks were identified with a signal to noise ratio of ∼8 (peak fluorescent intensity divided by the variation of the background), which resulted in a positional accuracy of ∼40 nm in the X- and Y-direction (determined by the quotient of the full-width-at-half-maximum of the Gaussian fit and the square root of the number of photons detected [38]). On average, each picture contained ∼1.5 peaks. Image sequences were recorded in series of 8 subsequent images with a time lag of either 6.25 ms or 12.5 ms (Figure 1C). Data on molecular dynamics were obtained for multiple step sizes. We used all time lags from 6.25 to 37.5 ms in our analysis. From each cell 180 series of 8 images were taken and data from 20 independent cells (imaged on at least 3 different days) was combined for the analysis. We used the Particle Image Correlation Spectroscopy (PICS) method to determine peak displacement over time [33]; explained in detail in Methods S1. PICS generates a cumulative probability function (P_cum_) of diffusion steps (characterized by *l*) for each time lag. P_cum_ can subsequently be fitted with a two population model:
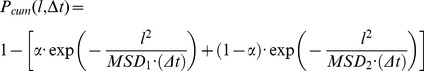
where MSD_1_ and MSD_2_ denote the mean square displacement of the first (fast) and the second (slow) fractions respectively, and α is the fraction size of the first (fast) fraction (Figure 1D). Although diffusion happens in 3D, we measure only the 2D projection, and to prevent distortion of the data due to molecules ‘escaping’ in 3D space, we restrict ourselves to only small time lags (up to 37.5 ms). This analysis was repeated for each time lag and α, MSD_1_ and MSD_2_ were plotted over time (Δt). All analyses were first performed on all data from each treatment group pooled together (n = 20). Subsequently, all analyses were run again in 3 fractions (n = 6/7) and these 3 separate analyses are used to generate standard errors of the mean [33]. Finally, OriginPro software was used to obtain weighted, linear fits, to calculate D_fast_ and D_slow_.

### FRAP

Before FRAP recordings, cells were exposed to 1 µM of the appropriate ligand for 3-6 hours in normal growth medium. For each experiment, a coverglass with transfected COS-1 cells was placed in a preheated ring and medium was replaced for empty D-MEM without phenol red, supplemented with 1 µM of the corresponding ligand. Cells were used for no longer than 90 minutes and kept at 37°C and 5% CO_2_. We used a Zeiss LSM510 META confocal laser scanning microscope equipped with a 40x/1.3NA oil-immersion objective, an argon laser (30 mW) and an AOTF. For FRAP analysis a narrow strip spanning the entire width of the nucleus was scanned at 514 nm excitation with short intervals (100 ms) at low laser power (0.2%). Fluorescence intensity was recorded using a 560-nm longpass filter. After 40 scans, a high intensity (100% laser power), 100 ms-bleach pulse at 514 nm was applied over the whole strip. Subsequently, the recovery of the fluorescence intensity in the strip was followed for another 55 seconds at 100 ms intervals. For each treatment group 30 cells were measured by FRAP on two separate days. All curves were normalized to baseline fluorescent intensity and combined. The FRAP data was quantitatively analyzed by comparing the experimental data to curves generated using a previously described Monte Carlo approach [6]. In short, the generated curve fitting best to the experimental curve (by ordinary least squares) was picked from a large set of computer simulated FRAP curves with a 3-population model, containing a diffusing fraction and two bound (immobile) fractions (Figure 1E). We take the D_fast_ obtained from SMM analysis as a fixed parameter in these simulation, leaving 4 parameters as variables: short bound fraction, long bound fraction (both ranging from 0–90%), and time spent in short and long bound state (ranging from 0.1 s to 1 s and from 1 s to 300 s respectively) (see also Methods S1). The parameters of the top 10 best fitting Monte Carlo curves were averaged to represent the properties of the fractions in the experimental data.

## Results

We first investigated the nuclear dynamics of the GR by SMM. We used COS-1 cells, transiently transfected with EYFP-tagged human GR (YFP-GR). This YFP-GR fusion protein was previously shown to retain a good transcriptional activity [3]. Before analysis, cells were exposed for 3 to 6 hours to a saturating dose (1 µM) of the high affinity GR agonist dexamethasone, which induces nuclear translocation of YFP-GR (Figure 1A). Nuclei were photobleached until single diffraction-limited fluorescence intensity peaks could be distinguished (Figure 1B). These peaks are attributed to single YFP-GR molecules as they had comparable width and intensity as fluorescence intensity peaks derived from single EYFP molecules previously observed using the same setup [37]. In our current approach, EYFP molecules were identified with a positional accuracy of ∼40 nm in one dimension (x or y). Next, GR mobility was analyzed by assessing molecule displacements over image sequences with short time lags (6.25 and 12.5 ms; Figure 1C), using the Particle Image Correlation Spectroscopy (PICS) analysis method [33]. We use PICS analysis instead of single particle tracking, as PICS is less affected by blinking of YFP or overlapping trajectories of multiple molecules [33], [34]. PICS analysis calculates the cumulative probability distribution for each displacement, which is subsequently fitted with multiple-population models (Figure 1D, see material and methods). DNA-bound and thus immobile molecules should show negligible displacement steps in these models. For YFP-GR, a one-population model was unable to describe the experimental data (Figure 1D), while a three-population model did not give consistent results over different time lags or resulted in two fractions with similar displacements. A two-population model fitted the observed displacements consistently, and with high accuracy, and was chosen for all analyses. Thus, we obtained the relative size and mean squared displacement (MSD) of two fractions of YFP-GR molecules that differed in their relative displacements over time.

**Figure 2:**
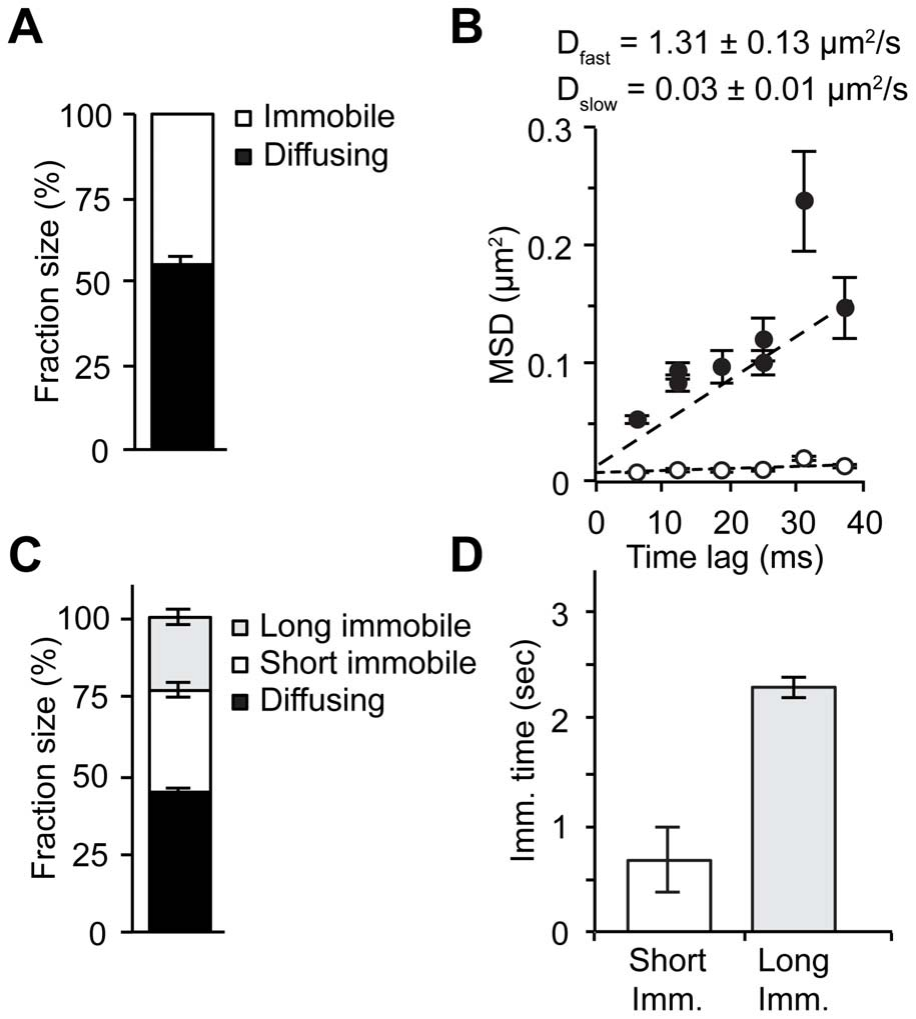
SMM and FRAP analyses provide a consistent model of the intranuclear mobility of the GR. (A) A two-population fit of SMM analysis for dexamethasone-bound YFP-GR identifies two fractions of approximately equal size. (B) Both fractions show a linear increase in mean squared displacement (MSD) over time, but with a 40-fold difference in MSD. Diffusion coefficients (D_fast_ and D_slow_) are calculated from a linear fit of the experimental data (dashed lines; D =  slope/4). The D_fast_ of 1.31 µm^2^/s fits to diffusing molecules, while the D_slow_ of only 0.03 µm^2^/s best fits to the slow movement of chromatin and the molecules bound to it. (C) A 3-population Monte Carlo simulation of the FRAP curve for dexamethasone-bound YFP-GR shows that half of the nuclear population is diffusing, while the remainder is subdivided into two bound fractions that differ in their immobilization times. The fraction size of the diffusing fraction is similar in size as that obtained from SMM analysis. (D) Both bound fractions are only transiently immobilized, with a 3-fold difference in duration. (A and B) Data represented as best fit ± SEM (of 3 separate PICS analyses). (C and D) Data represented as average of top 10% best fits ± SEM.

We plotted the MSDs of the two identified fractions versus the time lag and calculated the diffusion coefficients (D_fast_ and D_slow_; Figure 2B). The displacements of the “slow” fraction never exceeded our detection limit (0.009 µm^2^) by more than 2-fold and only increased marginally over time: D_slow_ of 0.03±0.01 µm^2^/s. This is very similar to the slow restricted movement of chromatin [31], [39], indicating that this “slow” fraction describes DNA-bound molecules. In contrast, the remaining fraction showed >40-fold higher displacements and a D_fast_ of 1.31±0.13 µm^2^/s, representing YFP-GR molecules diffusing through the nucleus. The nuclear GR population is approximately evenly distributed over the two fractions; 55.1±2.0% belongs to the diffusing fraction, which leaves 44.9±2.0% as bound fraction (Figure 2A, Table 1).

**Table 1:** Summary of all SMM and FRAP analyses of YFP-GR, YFP-MR and YFP-GR deletion mutants.

			SMM		FRAP	
Plasmid	Treatment	Fraction	Fraction size (%)	D (µm^2^/s)	Fraction size (%)	Imm. time (s)
**GR wt**	Δ-Flu	Diffusing	46.3±2.6	1.38±0.11	43±2.6	-
		Short	53.7±2.6	0.05±0.004	33±2.1	0.8±0.1
		Long			24±2.2	2.9±0.5
	Dex	Diffusing	55.1±2.0	1.31±0.13	44±2.2	-
		Short	44.9±2.0	0.03±0.009	33±2.6	0.7±0.1
		Long			23±2.6	2.3±0.3
	Predn	Diffusing	60.7±3.1	2.20±0.11	42±2.5	-
		Short	39.3±3.1	0.09±0.008	36±1.6	0.7±0.1
		Long			22±2.5	4.0±0.8
	Csol	Diffusing	55.6±3.5	1.77±0.10	58±2.0	-
		Short	44.4±3.5	0.04±0.003	19±3.8	0.5±0.1
		Long			23±2.6	2.0±0.0
	Cort	Diffusing	74.1±3.3	2.49±0.24	66±2.2	-
		Short	25.9±3.3	0.08±0.024	26±3.7	0.6±0.1
		Long			8±2.5	1.2±0.3
	RU486	Diffusing	69.1±2.4	2.86±0.11	66±1.6	-
		Short	30.9±2.4	0.14±0.018	24±3.1	0.5±0.1
		Long			10±2.6	1.4±0.3
**MR wt**	Aldo	Diffusing	54.1±3.4	1.43±0.04	45±1.7	-
		Short	45.9±3.4	0.05±0.002	32±2.0	0.8±0.1
		Long			23±2.1	2.9±0.5
	Cort	Diffusing	50.7±1.4	1.37±0.13	47±2.1	-
		Short	49.3±1.4	0.08±0.005	31±2.3	0.7±0.1
		Long			22±2.5	3.4±0.8
	Csol	Diffusing	51.5±0.8	1.96±0.19	44±2.2	-
		Short	48.5±0.8	0.05±0.002	32±2.9	0.6±0.1
		Long			24±2.7	2.3±0.3
	DOC	Diffusing	60.5± 3.6	1.60±0.13	57±1.5	-
		Short	39.5±3.6	0.06±0.003	23±3.0	0.6±0.1
		Long			20±3.1	2.3±0.3
	Dex	Diffusing	64.3±6.0	1.74±0.20	67±2.1	-
		Short	35.7± 6.0	0.04±0.003	22±4.4	0.7±0.1
		Long			11±3.1	1.7±0.5
	Spiro	Diffusing	78.8±2.3	2.71±0.05	71±3.5	-
		Short	21.2±2.3	0.06±0.018	23±3.7	0.5±0.1
		Long			6±1.6	1.2±0.3
	Epler	Diffusing	68.2±6.6	2.49±0.12	66±1.6	-
		Short	31.8±6.6	0.06±0.004	25±2.7	0.6±0.1
		Long			9±2.3	1.7±0.5
**GR ΔAF-1**	Dex	Diffusing	46.5±1.9	0.61±0.08	57±3.0	-
		Short	53.5±1.9	0.00±0.006	18±3.6	0.6±0.1
		Long			25±3.4	2.1±0.4
	Cort	Diffusing	64.7±2.8	2.69±0.08	62±2.5	-
		Short	35.3±2.8	0.05±0.012	27±4.0	0.6±0.1
		Long			11±2.3	1.6±0.3
**GR ΔDBD**	Dex	Diffusing	75.6±3.4	2.27±0.15	66±1.6	-
		Short	24.4±3.4	0.01±0.006	25±2.7	0.5±0.1
		Long			9±2.3	1.4±0.3
	Cort	Diffusing	81.3±1.0	2.37±0.19	79±3.1	-
		Short	18.7±1.0	0.06±0.004	18±3.9	0.5±0.1
		Long			3±1.5	0.6±0.3
**GR ΔLBD**	Dex	Diffusing	86.5±1.9	2.71±0.08	82±3.3	-
		Short	13.5±1.9	0.03±0.010	16±3.1	0.4±0.1
		Long			2±1.3	0.4±0.3

Short, ‘short’ bound fraction; long, ‘long’ bound fraction; D, diffusion coefficient; imm. time, average immobilization time; Δ-Flu, Δ-Fludrocortisone; dex, dexamethasone; Predn, prednisolone; csol, cortisol; cort, corticosterone; aldo, aldosterone; DOC, deoxycorticosterone; spiro, spironolactone; epler, eplerenone. Fraction size and diffusion coefficient for immobile fraction in SMM are for both immobile fractions combined. Results are represented as best fit ± SEM (of three separate fits) for SMM and as average ± SEM of top 10% fits for FRAP.

### FRAP analysis of dexamethasone-bound YFP-GR

Subsequently, we employed a quantitative FRAP approach on similarly treated YFP-GR expressing COS-1 cells. In selected nuclei a small strip, spanning the width of the nucleus, was bleached with a 100 ms pulse of maximal laser power. This bleached fluorescence within this area to ∼30% of baseline levels (Figure S1). The subsequent recovery of the fluorescence in this strip was recorded (with 100 ms intervals) for 55 seconds (Figure 1E). Comparable to previous results [3], [4], a complete recovery of YFP-GR fluorescence was seen well within 30 seconds (Figure 1E). The obtained recovery curves were quantitatively analyzed by fitting them to FRAP curves generated using Monte Carlo simulations [6], [35]. Our data was best fitted with a model in which freely diffusing molecules (diffusion rates as obtained by SMM were used) show transient binding with two different durations (‘short’ and ‘long’; Figure 1E). Quantitative FRAP analysis of dexamethasone-treated GR identified a diffusing fraction of 44±2%, a ‘short’ bound fraction of 33±3% (average binding of 0.7±0.1 sec) and a ‘long’ bound fraction of 23±3% (average binding of 2.3±0.3 sec) (Figure 2C and D).

As both bound fractions in FRAP remain bound for much longer time periods than the time range used in SMM (less than 50 ms), these two fractions could be distinguished using FRAP, but not by SMM. Indeed, the size of the single bound fraction in SMM, is similar to the combined size of the two bound fractions identified in FRAP (compare Figure 2A and 2C). Because we use the D_fast_ determined by SMM as a fixed parameter in the Monte Carlo simulations we wanted to make sure that this coefficient does not determine the distribution of the remaining fractions. Therefore, we redid the Monte Carlo modeling with the D_fast_±SEM, and this hardly affected the fraction sizes or immobilization times of the bound fractions (Figure S2). Thus, we conclude that the mobility patterns assessed by SMM at the millisecond range are confirmed with realistic accuracy using an independent FRAP approach.

### YFP-GR mobility is dependent on ligand structure

Next we used our combined SMM and FRAP approach to investigate how binding of different ligands affects GR-DNA binding dynamics. First, we showed that lowering the concentration of ligand increases the nuclear mobility of the GR. 1 µM and 100 nM of dexamethasone both induce a relatively immobile GR, but 10 nM (a concentration nearing the Kd of ∼5 nM [40], [41]) results in a smaller DNA-bound fraction and faster diffusion (Figure S3). Most likely this effect is due to an increased fraction of unbound GR within the nucleus. Without hormone, most of the GR remains in the cytoplasm. Still, the small fraction of nuclear GR that can be measured with SMM shows a very high mobility and an immobile fraction of only 16.6±5.0% (Figure S3). Previously, we showed by FRAP that the structure of the ligand is an important determinant of GR affinity also independent of the fraction of bound receptor (i.e. at above saturating concentrations) [3], [4]. We identified important roles for the 17-hydroxyl and 9-fluoro groups on the steroids, which induce a decrease in GR mobility. In the present study, this was studied in more detail in order to investigate which of the mobility parameters were affected. Therefore, we tested a panel of GR agonists that enabled us to study the effects of the 17-hydroxyl, 9-fluoro, and 16-methyl groups and the 1, 4-pregnadien structure of the A ring. We used dexamethasone (which contains all four structural elements), Δ-fludrocortisone (same structure as dexamethasone, but lacking the 16-methyl group), prednisolone (same structure as Δ-fludrocortisone, but lacking the 9-fluoro group), cortisol (same structure as prednisolone, but having a 4-pregnen instead of a 1, 4-pregnadien structure), and corticosterone (same structure as cortisol, but lacking the 17-hydroxyl group). In addition to this panel of agonists, the GR antagonist RU486 was used. Importantly, all hormones were administered at a saturating concentration (1 µM), thus the fraction of bound receptor should be similar for all ligands [4], [40], [42], [43].

**Figure 3:**
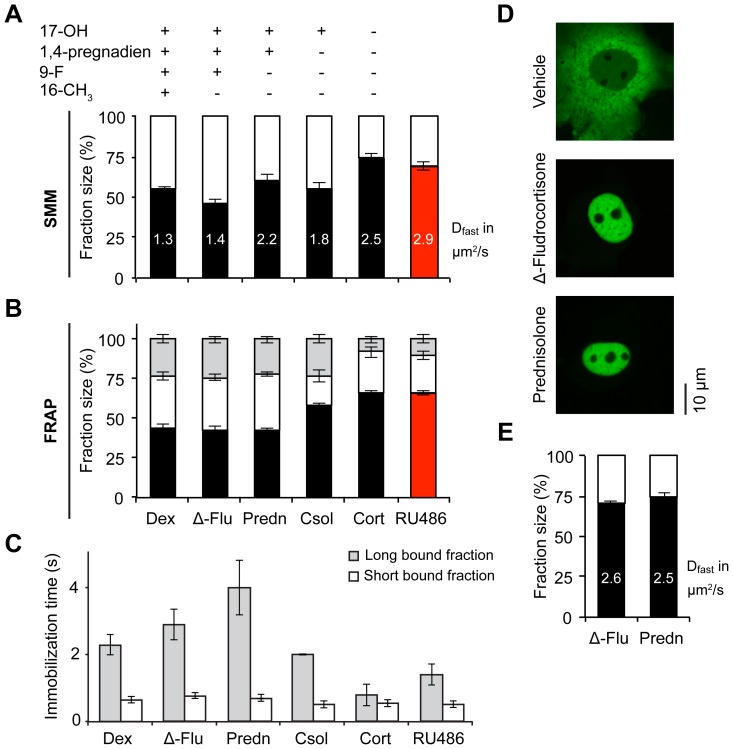
Ligand structure determines the nuclear mobility of the GR. A range of natural and synthetic agonists (black bars) and an antagonists (red bar) were tested for their effect on the intranuclear mobility of the GR by both SMM (A) and FRAP (B–C) analysis. Multiple structural elements of the steroids are associated with a reduced mobility of the receptor. Altered mobility can be reflected in all aspects of mobility: a larger bound fraction (SMM; white bars and FRAP; white and light grey bars combined) a lower diffusion coefficient (in µm^2^/s, written in its corresponding bar in A) and longer immobilization times (C). (D and E) A mutation of phenylalanine 623 to alanine (F623A) prevents interactions of the 9-fluoro group of steroids within the ligand binding pocket of the GR. F623A YFP-GR still translocates completely to the nucleus after 3 hours of 1 µM prednisolone or Δ-fludrocortisone treatment (D). SMM analyses of nuclear F623A YFP-GR kinetics shows that the mobility of F623A YFP-GR is highly similar after either Δ-fludrocortisone or prednisolone treatment (black bars for the diffusing fraction, with their corresponding diffusion coefficient (in µm^2^/s) written within their corresponding bar; (E)). SMM: n = 20, FRAP: n = 30. Data represented as total fit ± SEM (of 3 separate PICS analyses) for SMM and as average of top 10% fits ± SEM for FRAP. Δ-flu; Δ-fludrocortisone, dex; dexamethasone, Predn; prednisolone, csol; cortisol, cort; corticosterone. The data for GR-dexamethasone is the same as in Figure 2.

Again, the two independent experimental approaches gave a consistent pattern of fraction sizes for all 6 ligands tested. On average the size of the diffusing fractions identified with SMM and FRAP differed by only 7.8±2.6% (Table 1). Although a few inconsistencies occurred, the data show that the 16-methyl group does not affect GR mobility, but that the other structural elements decrease the mobility of the receptor, indicating increased DNA binding (Figure 3A and B).This decreased mobility was reflected in changes in one or more parameters measured, but the data indicate that ligand structure may have effects on all parameters measured. Both the size of the bound fractions and their respective binding times were different between ligands, so both on- and off-rates of DNA binding were altered. In addition, the diffusion coefficient of the diffusing fraction was affected, indicating that altered DNA binding is associated with changes in diffusion of the receptor (Figure 3A and Table 1). Binding of the antagonist RU486 induces a very mobile nuclear YFP-GR, which is comparable to the effect of corticosterone (Figure 3A and B).

It is known that the 9-fluoro group (present on Δ-fludrocortisone and dexamethasone) creates a strong hydrogen bond with phenylalanine at position 623 of GR's ligand binding pocket [44], suggesting that this amino acid is crucial in conferring the effects of the 9-fluoro group. To test this association, phenylalanine 623 was mutated to an alanine (F623A). We tested the mobility of F623A with SMM in the presence of prednisolone and Δ-fludrocortisone, which are identical except that Δ-fludrocortisone contains a 9-fluoro group and prednisolone does not. In the presence of either steroid the F623A mutant fully translocates to the nuclear compartment (Figure 3C). Within the nucleus, no difference in F623A mobility was observed between Δ-fludrocortisone and prednisolone (Figure 3D, compare to data in Figure 3A). Therefore we conclude that the effect of the 9-fluoro group on DNA binding dynamics is indeed mediated by phenylalanine 623.

### YFP-MR mobility is also dependent on ligand structure

**Figure 4:**
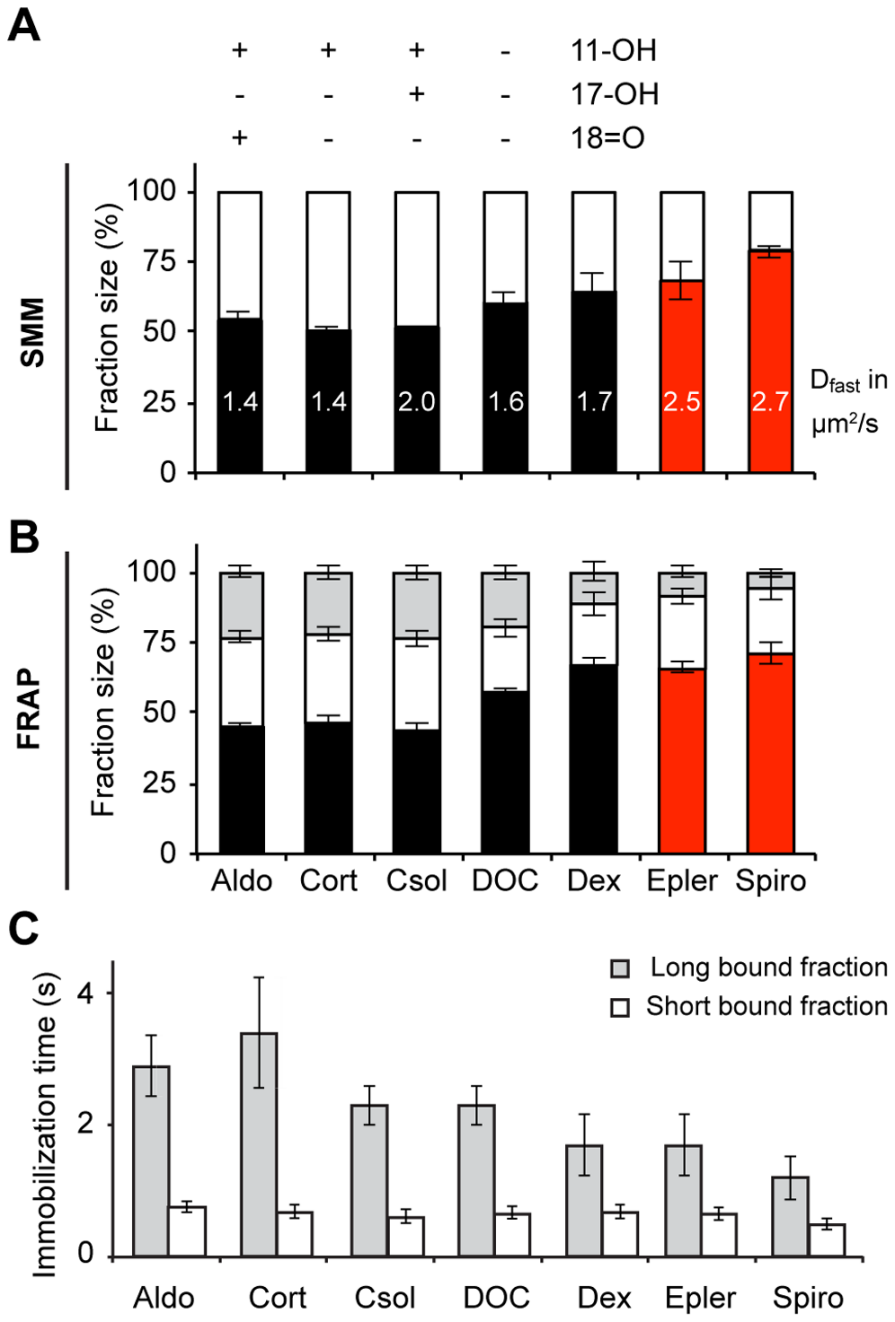
Ligand structure determines the nuclear mobility of the MR. A range of natural and synthetic agonists (black bars) and antagonists (red bars) were tested for their effect on the intranuclear mobility of the MR by both SMM (A) and FRAP (B–C) analysis. The MR and GR share several agonists, but the binding and functional characteristics differ. Indeed, a weak agonist for the GR, corticosterone (cort), which gave a very mobile GR, instead leads to a low mobility for the MR. A large bound fraction (SMM; white bars and FRAP; white and light grey bars combined) a low diffusion coefficient (in µm^2^/s, written within its corresponding bar in A) and long immobilization times (C). Of all functional steroid side groups, only the 18-keto (18 = O) group appears to affect the mobility of the MR. SMM: n = 20, FRAP: n = 30. Data represented as total fit ± SEM (of 3 separate PICS analyses) for SMM and as average of top 10% fits ± SEM for FRAP. Aldo; aldosterone, csol; cortisol, DOC; deoxycorticosterone, dex; dexamethasone, epler; eplerenone, spiro; spironolactone.

The mineralocorticoid receptor (MR) is a steroid receptor with high similarity to the GR. Not only is it structurally related to the GR, but it is also activated by some of the same ligands, with different affinities [43], [45], [46], [47]. For example, corticosterone is a high-affinity agonist for the MR, while dexamethasone has only a moderate affinity for the MR [42], [47], and the reverse is true for the GR. To elucidate whether agonist effects on receptor DNA binding dynamics result from specific ligand-receptor interaction or from ligand-specific characteristics, we tested the intranuclear dynamics of YFP-MR after activation by a selected panel of agonists and antagonists. A panel was tested that enabled us to study the effects of the 18-keto, and 11- and 17-hydroxyl groups on naturally occurring mineralocorticoid receptor agonists. We used aldosterone (which contains an 18-keto and 11-hydroxyl group), corticosterone (same structure as aldosterone, but lacking the 18-keto group), cortisol (same structure as corticosterone, but containing an additional 17-hydroxyl group), and deoxycorticosterone (same structure as corticosterone, but lacking the 11-hydroxyl group). In addition, the GR agonist dexamethasone and two MR antagonists, spironolactone and eplerenone were included. The results are presented in Figure 4.

Upon binding of corticosterone, 50.7±1.4% (SMM) to 47±2% (FRAP) of nuclear MR molecules belong to the diffusing fraction, with a D_fast_ of 1.37±0.13 µm^2^/s. The remaining 53% to 49.3% of the nuclear MR population is bound to DNA, for 0.7±0.1 second (31±2.3%) or 3.4±0.8 seconds (22±2.5%, Figure 4). This pattern closely resembles that of the GR after activation by the agonists dexamethasone and Δ-fludrocortisone, and is very different from the pattern observed for GR in the presence of corticosterone, confirming that the ligand-receptor interaction determines receptor-DNA binding and not the nature of the ligand. Aldosterone and cortisol induced a similar mobility of the MR as corticosterone does, which indicates that the 11- and 17-hydroxyl groups are not involved in determining MRs DNA binding dynamics. In contrast, deoxycorticosterone gave a higher nuclear mobility, which suggests that the presence of the 11-hydroxyl group positively affects DNA binding of MR. As expected, the GR agonist dexamethasone and the antagonists spironolactone and eplerenone induced a very mobile receptor (Figure 4). Similarly to YFP-GR, a higher mobility could be reflected in all parameters tested: the size of the bound fractions, binding times and the diffusion coefficient.

### Specific receptor domains determine YFP-GR mobility

**Figure 5:**
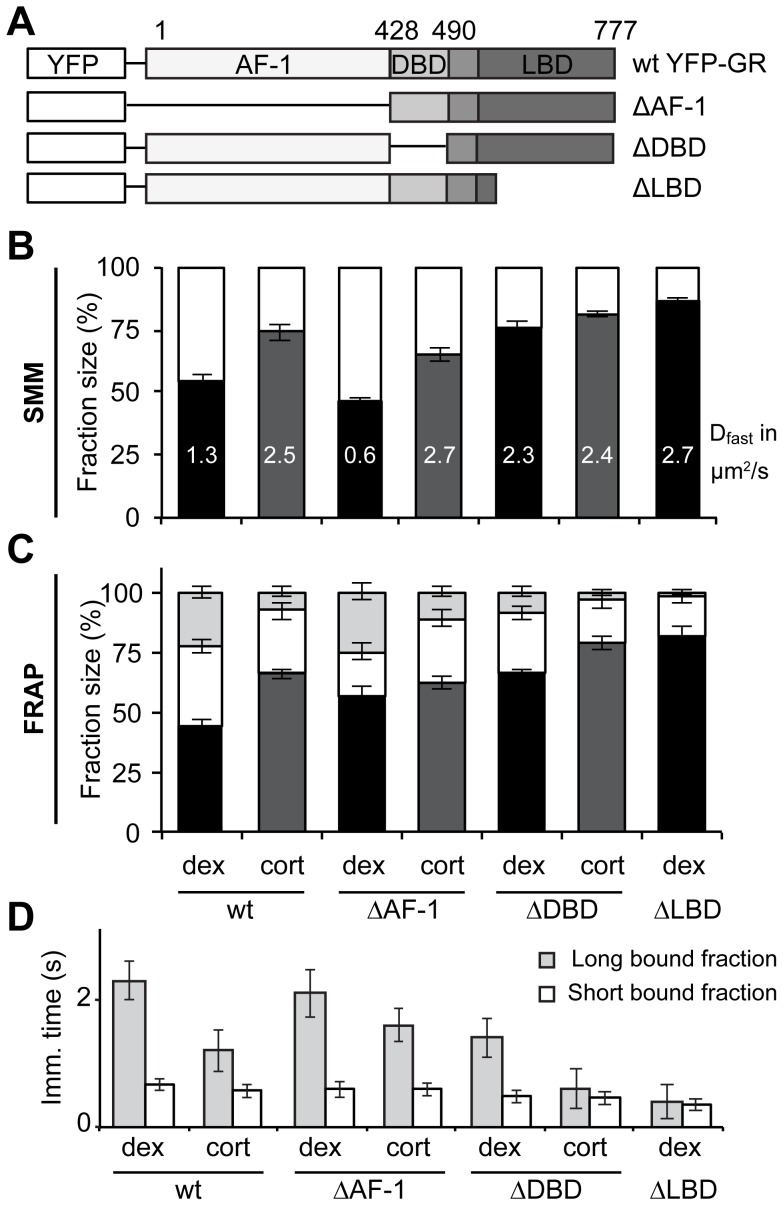
Loss of either the DNA-binding or the ligand-binding domain results in a high GR mobility. (A) Schematic representation of three functional YFP-GR deletion mutants tested. (B and C) Fraction distributions as analyzed by SMM (B) and FRAP (C). Diffusion coefficients are written within the corresponding bars in B (in µm^2^/s). (D) Immobilization times of both bound fractions in FRAP. While loss of the AF-1 domain hardly affects GR's nuclear mobility, deletion of the DBD and especially the LBD leads to a very mobile receptor with reduced frequency and average duration of DNA-binding and a higher diffusion coefficient. SMM: n = 20, FRAP: n = 30. Data represented as total fit ± SEM (of 3 separate PICS analyses) for B and as average of top 10% fits ± SEM for C and D. The data for wild type GR is the same as in Figure 3.

In order to elucidate the role of the different domains of GR on DNA-binding dynamics, we tested three different GR deletion mutants, each lacking one of its functional domains. We used YFP-GR ΔAF-1 (lacking the N-terminal domain containing the AF-1 (amino acids 9-385)), YFP-GR ΔDBD (lacking the DNA-binding domain (amino acids 428–490)) and YFP-GR ΔLBD (lacking the ligand-binding domain (amino acids 551–777)), see Figure 5A. We investigated the nuclear dynamics of the three deletion mutants of YFP-GR by SMM and FRAP in the presence of dexamethasone or corticosterone. All results are shown in Figure 5. Deletion of the AF-1 showed the smallest effect on DNA binding dynamics of the receptor. Dexamethasone binding to the ΔAF-1 mutant induces a large DNA-bound fraction and long binding times, and a slow diffusing fraction. In contrast, corticosterone binding results in a much faster receptor with less stable DNA binding (Figure 5B and C). Thus, without its N-terminal domain, the GR's intranuclear mobility is still differently affected by high and low affinity agonists, and its DNA binding dynamic is similar to the wild type receptor.

As expected, deletion of the DBD did affect the receptor's DNA binding dynamics (Figure 5B and C). For corticosterone-bound GR, deletion of the DBD slightly increased the size of the diffusing fraction and completely prevents the longer binding events, resulting in two bound fractions with almost equal immobilization times: 0.5±0.1 s (18±3.9%) and 0.6±0.3 s (3±1.5%, Figure 5C). For dexamethasone-bound ΔDBD not all stable DNA-binding is lost; here 25±2.7% remains immobilized for 0.5±0.1 s and even 9±2.3% remains immobilized for 1.4±0.3 s. Dexamethasone-bound YFP-GR ΔDBD does show a large increase of the size of the diffusing fraction (from 44–55% (wild type) to 76–66% (ΔDBD)), and a ∼2-fold higher diffusion coefficient (Figure 5B and C). Thus, deletion of the DBD induces less frequent and shorter immobilizations for both dexamethasone and corticosterone bound GR, but a fraction of longer bound YFP-GR ΔDBD remains when bound to dexamethasone. Deletion of the DBD abolishes all direct binding of the GR to the DNA, but it has been shown that a large fraction of GR binding sites does not contain a GRE (estimates range between 40 and 75% [48], [49], [50]. Binding to these sites therefore requires interaction with other factors. An intact DBD is required for physical interaction with a variety of transcription factors, like NF-κB [51] and T-bet [52]. It can however not be ruled out that GR is able to bind to DNA sites through (direct or indirect) interaction with factors that have not been studied in detail and are independent of the presence of a DBD function. For example, several novel interaction partners for GR have recently been identified in neuronal cells [48].

Deletion of the LBD prevents the ligand-induced conformational change that is required for any type of stable interaction with DNA. As expected, YFP-GR ΔLBD was the most mobile receptor variant, it had the smallest DNA-bound fraction (13.5% to 18% in SMM and FRAP respectively) with a single (short) binding state of 0.4±0.1 s and a high diffusion coefficient (2.71±0.08 µm^2^/s; Figure 5B and C). Most importantly, this mutant did not show a stably-bound fraction. Although the ΔLBD mutant has been shown to induce transcription from GRE-containing promoters that have transiently been transfected into cells [53], it has been demonstrated that this mutant is not able to stimulate transcription from native chromatin templates [54]. Our results indicate that this latter effect is due to the absence of stable DNA binding of this mutant in living cells.

### YFP-GR mobility is stable across cell lines and expression levels

**Figure 6:**
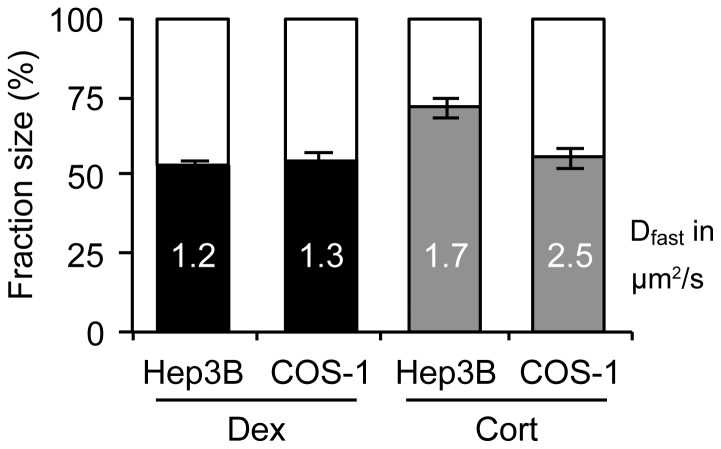
A similar pattern of YFP-GR's nuclear mobility in stably transfected Hep3B cells. SMM analysis of YFP-GR's nuclear mobility after treatment (3–6 h with 1 µM) with either dexamethasone or corticosterone was performed in Hep3B cells stably transfected with YFP-GR. These experiments were performed to check for effects of differences in cellular context and a lower level of YFP-GR expression on the mobility patterns. Both the size of the diffusing fraction (filled bars) and the diffusion coefficients (written in their corresponding bars in µm/s^2^) were highly similar between COS-1 and Hep3B cells. COS-1 data is the same as in Figure 2. All groups: n = 20. Data is represented as total fit ± SEM (of 3 separate PICS analyses).

In order to test whether overexpression or transient transfection had produced artifacts in our experiments, we stably transfected Hep3B cells with the same YFP-GR expression vector. The resulting cell line showed a much lower expression level of YFP-GR than that observed in the transiently transfected COS-1 cells. The DNA-binding dynamics were studied of corticosterone- and dexamethasone-bound YFP-GR in this cell line with SMM. Dexamethasone induced a diffusing fraction of 52.9±1.6% and a diffusion coefficient of 1.16 µm^2^/s±0.08 in Hep3B cells (Figure 6). As expected, corticosterone treatment induced a more mobile YFP-GR, with a diffusing fraction of 71.6±3.4% and a D_fast_ of 1.70±0.16 µm^2^/s (Figure 6). These results were very similar to those obtained in COS-1 cells, indicating that our results are not cell-type specific or affected by expression levels obtained by transient transfection.

## Discussion

Here we report on a combination of single-molecule microscopy (SMM) and quantitative FRAP analysis to characterize the intranuclear dynamics of the GR. In our SMM experiments, we find that single molecules of nuclear YFP-GR can be detected with high spatial and temporal resolution and that by subsequent data analysis two fractions of GR molecules are detected; a diffusing and a (DNA-)bound fraction. For all 18 treatment groups studied, this two-population model consistently fitted the experimental data with high accuracy. To enable cross-validation with an established technique, we combined our SMM analysis with a second technique, FRAP. We analyzed the FRAP curves using established Monte Carlo simulations [6], [36]. To best describe the FRAP recovery curves we required two bound fractions (for most receptors), which differed 2-4 fold in their binding duration. The binding times of both bound fractions are orders of magnitude longer than the time scale used in our SMM experiments and these fractions combined represent the single bound fraction detected in SMM, providing two independent estimates of the size of this (combined) fraction. Within our 18 experimental conditions, the sizes of the combined bound fractions determined by SMM and FRAP showed an average difference of only 6.5±1.1%. This high level of consistency between the two independent techniques shows that a combination of techniques generates a reliable quantitative description of protein dynamics.

Combinations of FRAP and fluorescence correlation spectroscopy (FCS) have been reported earlier [19], [20]. Here, FCS and FRAP generally gave comparable estimates, although large discrepancies were found for binding times, due to laser irregularities. Recently, Mazza and colleagues reported on a similar combinational approach with FRAP and single-molecule microscopy, in their case also combined with FCS [19]. In this study the mobility of p53, a well-known transcription factor was assessed and single-molecule tracking was used to guide the choices in models used for FRAP and FCS quantitation. Wild type p53 showed a much smaller DNA-bound fraction (∼20%) than agonist-activated GR does in our study, but in both studies mutations in the DNA-binding domains give a large reduction in size and residence time of the DNA-bound fractions [19]. Similarly, another study investigated the nuclear dynamics of the transcription factor STAT1 by single-molecule tracking [22]. Activation of STAT1 by its activator (interferon-γ) resulted in a large increase in both the size of the bound fraction and binding duration. In a third study, Gebhardt et al. [18] applied SMM on the GR, using reflected light sheet microscopy technology with a temporal resolution and positional accuracy comparable to our study. In this study unbound and dexamethasone-bound GR and the ΔDBD mutant were analyzed. Their data are well in line with ours, in particular the obtained values for the sizes of the diffusing and bound fractions and of binding times [18]. Discrepancies exist in the analysis of the diffusing fraction. Gebhardt et al. studied displacements during a fixed time interval of 10 ms, and could fit the distribution of these displacements using a model with two freely diffusing fractions. In the present study, displacements were investigated using a series of eight increasing time intervals. Although at some time intervals a third intermediate fraction could be distinguished, the size and diffusion rate of this fraction were not consistent between time intervals. We therefore fitted our data to a model containing only one freely diffusing fraction. As shown by Mazza et al. [19], it is likely that any diffusion coefficient is a simple representation of the more complex nature of transcription factor diffusion on a continuous scale. Importantly, Mazza et al. [19] also showed that since the DNA-bound fraction and the freely diffusing fraction are in general well separated, the determination of the size of the DNA-bound fraction and its binding time is not very sensitive to details of the method used for analysis of the freely diffusing fraction.

### Ligand structure affects the DNA-binding profile of nuclear GR

We observed profound differences in the nuclear dynamics of the GR and MR depending on the ligand it was bound to (Figures 3 and 4), even among agonists. For example, the synthetic GR agonists dexamethasone and Δ-fludrocortisone induce a larger DNA-bound fraction with longer residence times than the naturally occurring agonists cortisol and corticosterone. Structure-function studies showed that the 17-hydroxyl, and 9-fluoro groups and the 1,4-pregnadien structure of the A ring of these steroids were involved in the increased DNA binding of GR. For MR, different structural elements appear to play a role (the 11-hydroxyl group increases the frequency and duration of DNA binding of MR), which demonstrates that it is the interaction between the steroid and the receptor that determines its mobility. To confirm this, we showed that the effect of the 9-fluoro group depends on the presence of phenylalanine at position 623 of the GR LBD, the amino acid it is known to interact with [44]. This phenylalanine residue, like the glutamine residue at position 642 which interacts with 17-hydroxyl group, is located in a region of the LBD that has been shown to be involved in receptor dimerization [44]. It may therefore be suggested that these specific interactions shape the receptor into a conformation that favors receptor dimerization, and that these dimers have higher DNA binding affinity. We have previously suggested a similar model for AR dimerization and DNA binding [2].

Many of these structural elements also affect the affinity of the ligand and it could therefore be argued that the affinity of the ligand determines the receptor mobility. However, affinity and mobility are not always correlated. In the present study, we show that the 11-hydroxyl group affects the mobility of the MR (there is a difference between the effects of corticosterone and deoxycorticosterone), but it has been shown not to alter the receptor binding affinity [55]. In addition, in a previous study we have shown that the 16-hydroxyl group of triamcinolone dramatically decreases the binding affinity for GR, but leaves GR mobility unaffected [3], [4]. Furthermore, mechanistically it is unlikely that ligand affinity is a determinant of receptor mobility since all ligands are administered at a saturating concentration [3]. Moreover, ligand dissociation rates are in the order of minutes (corticosterone) to hours (dexamethasone) [56], [57], whereas the immobilizations of the receptor observed in this study are in the order of seconds.

### A model of GR-DNA interactions

Interestingly, our data shows a strong correlation between different components of the mobility pattern. Immobilization times correlate to the size of the bound fractions, thus both on and off rates of DNA binding are affected. More surprisingly, we also found that a low frequency and duration of binding events correlated with a higher diffusion coefficient throughout our different experiments. Thus, where antagonist-bound or low-affinity agonist bound GR and MR, and the ΔDBD and ΔLBD GR mutants were all associated with a low frequency of DNA-binding, these same receptors generally showed a high diffusion coefficient (1.5 to 2-fold higher than that of highest-potency agonist bound MR or GR, see Table 1). This suggests that all components of the mobility pattern are associated with each other and presumably are representations of a same biological phenomenon, i.e. DNA-binding. A plausible explanation could be that changes in the diffusion coefficient are due to DNA-binding events shorter than the temporal resolution of our SMM experiments (∼6 ms), which result in a decreased effective diffusion coefficient as long as the system is in equilibrium [58]. Alternatively, reduced diffusion coefficients can be caused by an increased size of the diffusing protein complex (e.g. through increased co-factor binding affinity).

Thus, for agonist-bound GR (and MR) we identified three possible DNA-binding events: frequently in a very transient manner (<6 ms), intermitted with transient binding (∼0.5 s) and occasionally more stable interactions (>1 s). This fits well with the idea that steroid receptors and other transcription factors search the DNA by different forms of low affinity DNA interactions and are only occasionally bound for longer time periods at their high-affinity target sites. Indeed, steroid receptors do not show competition for high-affinity binding sites, and in fact seem to do the opposite (assisted loading), suggesting that high-affinity DNA-binding cannot make up a large population [59]. Multiple *in vitro* studies and theoretical modeling approaches have suggested that frequent low-affinity interactions with DNA increase the efficiency of transcription factor target finding, because it keeps the transcription factor in close proximity of open DNA [31], [60], [61]. We suggest that the more transient interactions identified in our quantitative analysis represent non-specific DNA binding and that the longest DNA-binding events represent specific DNA binding.

Binding times for this specific DNA binding varied for GR between ∼1 and ∼4 seconds, depending on the ligand. The relationship between the time an individual receptor is bound to a target site and its effect on gene transcription remains to be established. Individual receptors shuttle on and off the DNA and a large variety of cofactors is attracted in a dynamic fashion as well. This results in a highly dynamic multi-protein complex interacting with a target promoter [62], which is involved in chromatin remodeling and modification in addition to transcriptional activation. A higher stability of this complex, reflected in a longer binding time of the receptor, may facilitate these processes, which may ultimately lead to an increased level of gene transcription.

## Supporting Information

Figure S1 **FRAP on life and fixed cells.** FRAP was performed on live and fixed (120 min in 4% PFA) cells, both expressing wild type GR and treated with 1 µM dexamethasone. Bleaching efficiency is similar between live and fixed cells, and no further bleaching during the recovery phase was observed in the fixed cells. *n = 30*.
(TIF)

Figure S2 **No major effect of error in diffusion coefficient on FRAP parameters.** The diffusion coefficient (D_fast_) obtained by SMM was used as input parameter in the Monte Carlo simulationss of the FRAP experimnets. Here we investigate whether small alterations in the D_fast_ affect the remaining FRAP parameters. The Monte Carlo modeling of wild type GR treated with 1 µM dexamethasone was performed with the D_fast_ – SEM, D_fast_ and D_fast_ + SEM. All diffusion coefficients gave a good fit of the raw FRAP curve (A). Only subtle differences in the fraction distribution (B) and immobilizations times of both fractions (C) were seen and no relationship between D_fast_ and any of the tested parameters could be established. *n = 30, data is represented as average of top 10% fits ± SEM*.
(TIF)

Figure S3 **Nuclear dynamics of the GR are dependent on ligand concentration.** In order to investigate the effect of ligand concentration on GRs nuclear dynamics, wild type GR expressing COS-1 cells were treated with 0 nM (vehicle), 10 nM, 100 nM or 1000 nM of dexamethasone for 3 hours and measured by SMM. Increasing the concentration from 10 nM to 100 nM dexamethasone leads to a decreased motility of the receptor as witnessed by a larger immobile fraction (A) and smaller displacements of the diffusing fraction (B). Increasing the concentration of dexamethasone to 1000 nM does not further affect the receptors dynamics; suggesting that the effect is saturated. Vehicle treated GR remains very dynamic within the nucleus, with a small immobile fraction and large displacements. *n = 15*–*20, only 6.25 ms time lags were measured. Data represented as total fit ± SEM (of 2*–*3 separate PICS analyses). Due to the small amount of GRs translocating to the nucleus in the vehicle condition, only the first two displacements step could be reliably assessed.*
(TIF)

Methods S1 **Supplementary methods.**
(DOCX)
