# Supplementary figures and images for: Quantitation of Glucocorticoid Receptor DNA-Binding Dynamics by Single-Molecule Microscopy and FRAP
Source: PLoS One. 2014 Mar 14;9(3):e90532. doi: 10.1371/journal.pone.0090532 (PMC3954550; doi:10.1371/journal.pone.0090532)

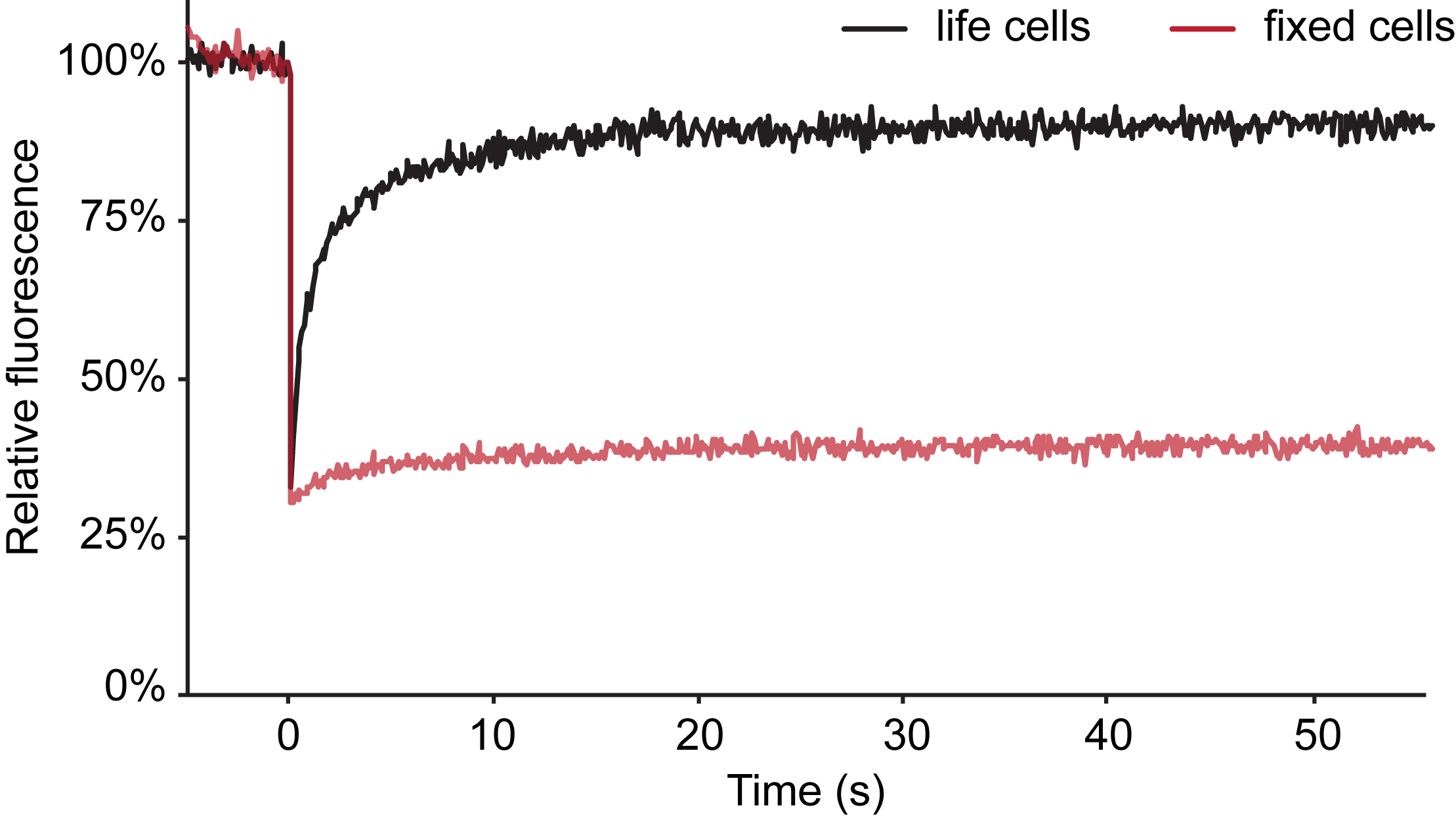

Supplement: Figure S1 — FRAP on life and fixed cells. FRAP was performed on live and fixed (120 min in 4% PFA) cells, both expressing wild type GR and treated with 1 µM dexamethasone. Bleaching efficiency is similar between live and fixed cells, and no further bleaching during the recovery phase was observed in the fixed cells. n = 30. (TIF) [file pone.0090532.s001.tif]

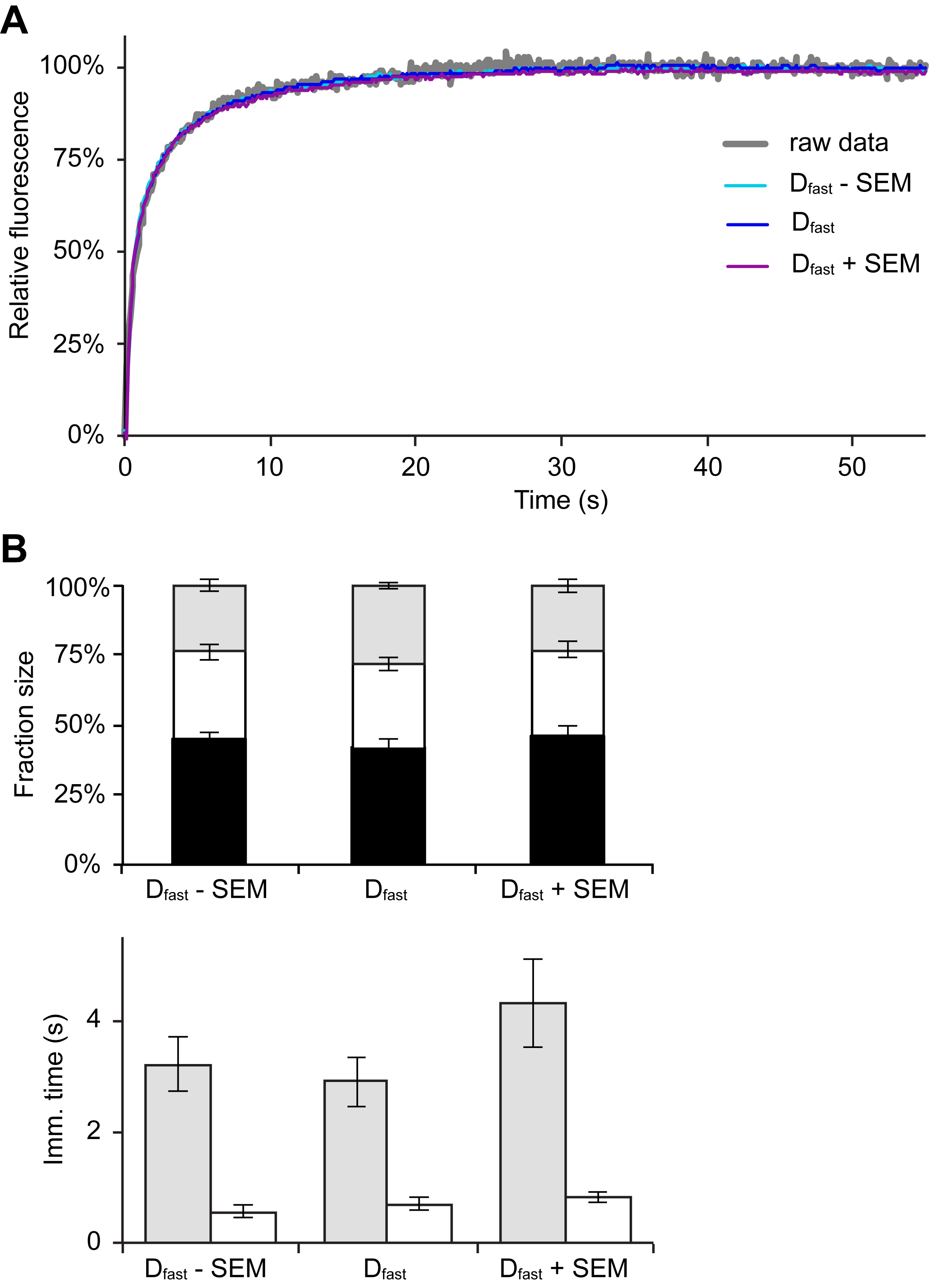

Supplement: Figure S2 — No major effect of error in diffusion coefficient on FRAP parameters. The diffusion coefficient (Dfast) obtained by SMM was used as input parameter in the Monte Carlo simulationss of the FRAP experimnets. Here we investigate whether small alterations in the Dfast affect the remaining FRAP parameters. The Monte Carlo modeling of wild type GR treated with 1 µM dexamethasone was performed with the Dfast – SEM, Dfast and Dfast + SEM. All diffusion coefficients gave a good fit of the raw FRAP curve (A). Only subtle differences in the fraction distribution (B) and immobilizations times of both fractions (C) were seen and no relationship between Dfast and any of the tested parameters could be established. n = 30, data is represented as average of top 10% fits ± SEM. (TIF) [file pone.0090532.s002.tif]

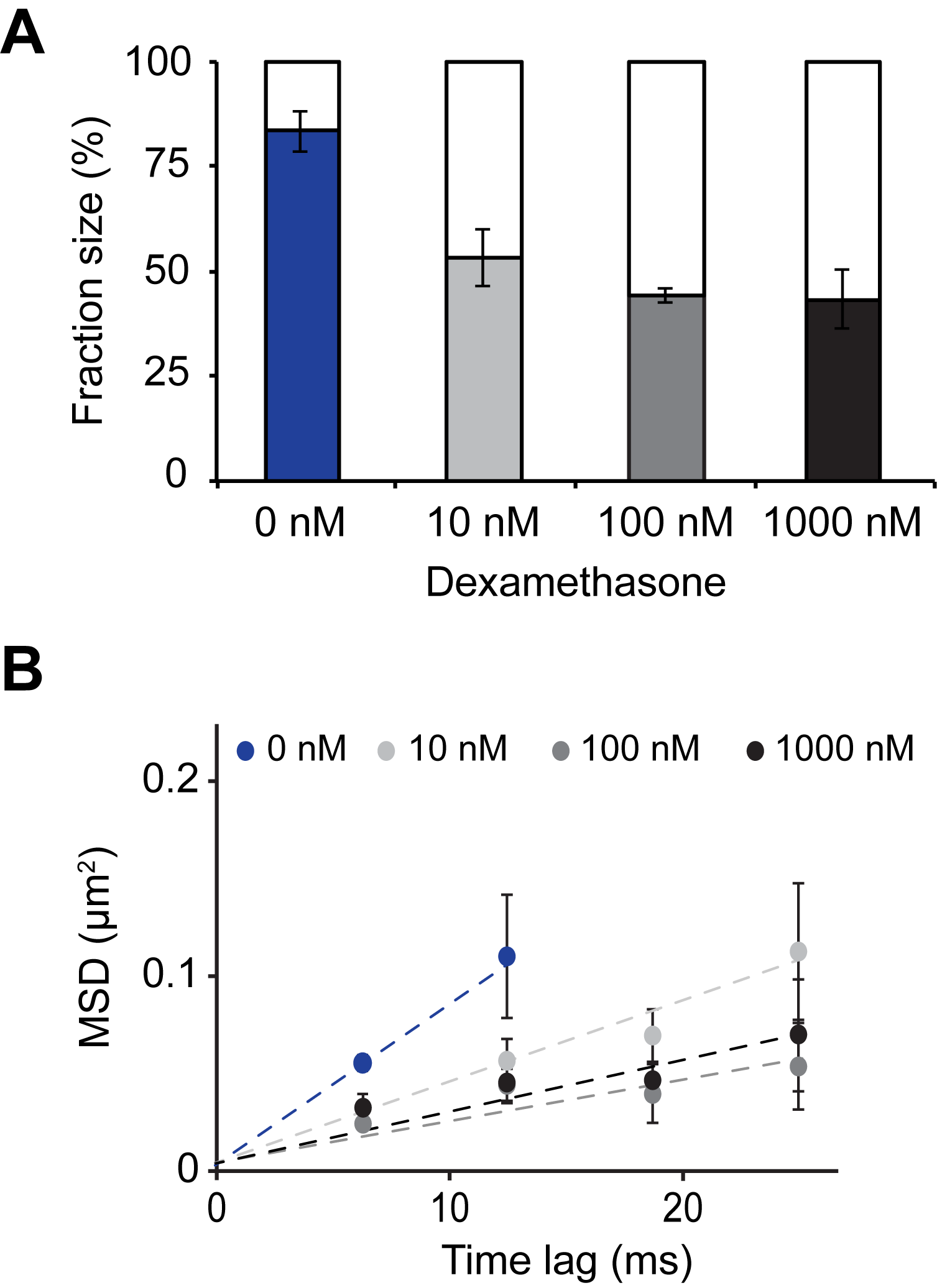

Supplement: Figure S3 — Nuclear dynamics of the GR are dependent on ligand concentration. In order to investigate the effect of ligand concentration on GRs nuclear dynamics, wild type GR expressing COS-1 cells were treated with 0 nM (vehicle), 10 nM, 100 nM or 1000 nM of dexamethasone for 3 hours and measured by SMM. Increasing the concentration from 10 nM to 100 nM dexamethasone leads to a decreased motility of the receptor as witnessed by a larger immobile fraction (A) and smaller displacements of the diffusing fraction (B). Increasing the concentration of dexamethasone to 1000 nM does not further affect the receptors dynamics; suggesting that the effect is saturated. Vehicle treated GR remains very dynamic within the nucleus, with a small immobile fraction and large displacements. n = 15–20, only 6.25 ms time lags were measured. Data represented as total fit ± SEM (of 2–3 separate PICS analyses). Due to the small amount of GRs translocating to the nucleus in the vehicle condition, only the first two displacements step could be reliably assessed. (TIF) [file pone.0090532.s003.tif]
