# Supplementary material for: Quantitation of Glucocorticoid Receptor DNA-Binding Dynamics by Single-Molecule Microscopy and FRAP
Source: PLoS One. 2014 Mar 14;9(3):e90532. doi: 10.1371/journal.pone.0090532 (PMC3954550; doi:10.1371/journal.pone.0090532)
Supplement: Methods S1 — Supplementary methods. (DOCX) [file pone.0090532.s004.docx]

**Supplementary methods**

COS-1 cells were grown in high-glucose D-MEM (Invitrogen) supplemented with 10% FBS and 1% penicillin/streptomycin (both GIBCO). 24 h prior to transfection, cells were plated on sterile coverglasses (25 mm diameter). Cells were transfected with the TransIT-COS kit (Mirus) according to the manufacturer’s instructions at a concentration of 500 ng DNA/10 cm^2^. Transfected cells were used in experiments 2-5 days after transfection. For one experiment, Hep3B cells were used, stably transfected with the pEYFP-hGR expression vector (1). These cells were grown in α-MEM (Cambrex), supplemented with 5% FBS, 2 mM L-glutamine, 1% Penicillin/Streptomycin and 600 µg/ml G418 (Invitrogen). The plasmid pEYFP-hMR was generated by PCR amplification (Phusion HF polymerase, Finnzymes) of the human MR gene from a pRSV human MR template (kindly provided by Dr. R. Evans (gene expression laboratory and HHMI, The Salk Institute for Biological Studies, La Jolla, CA). A set of primers was designed to generate BglII and SmaI sites at the 5’ and 3’ end of the MR coding sequence, with primer sequences: (FW: GCAGAAGATCTGGAGCAGGTGCTGGAGCAGGTGCTGGAGCAGGTGCTATGGAGACCAAAGGCTACCA and RV: GACCCGGGTGTGGAACAACACAGGGAAA). Subsequently, the PCR fragment was digested with BglII and SmaI, purified and cloned into the pEYFP-C1 vector, resulting in a vector with an in-frame fusion of hMR with EYFP, separated by 17, mostly nonpolar, amino acids. Plasmid integrity was checked by sequencing.

**Compounds.** The following ligands were used in these studies: dexamethasone, corticosterone, cortisol, Δ-fludrocortisone (1,4-pregnadien-9α-fluoro-11β,17,21-triol-3,20-dione), prednisolone (1,4-pregnadien-11β,17,21-triol-3,20-dione), RU486 (4,9-estradien-17α-propynyl,11β-[4-dimethylaminophenyl]-17β-ol-3-one), aldosterone, deoxycorticosterone **(21-hydroxy-4-pregnene-3,20-dione, 4-pregnen-21-ol-3,20-dione),** spironolactone **(4-pregnen-21-oic acid-17α-ol-3-one-7α-thiol γ-lactone 7-acetate, 7α-(acetylthio)-17α-hydroxy-3-oxopregn-4-ene-21-carboxylic acid γ-lactone)** and eplerenone (pregn-4-ene-7,21-dicarboxylic acid, 9,11-epoxy-17-hydroxy-3-oxo-, gamma-lactone, methyl ester). All steroids were purchased from Sigma-Aldrich and diluted in 100% EtOH to a concentration of 1 mM except for eplerenone, which was diluted in DMSO. All steroids were used at a final concentration of 1 μM in the medium.

**PICS analysis of single-molecule kinetics.** In PICS, the cross-correlation between peak positions at two different time lags (for example t = 0 ms and t = 6.25 ms) is calculated. This yields the cumulative probability distribution (C_cum_) of all ‘diffusion steps’ detected within 6.25 ms. C_cum_ includes both contributions from diffusing molecules as well as random correlations between unrelated molecules in the two frames. The latter follows a linear relation in the cumulative plot and was subtracted prior to further analysis (2). From the remaining cumulative probability function (P_cum_) of diffusion steps *l*, we use population modeling to calculate diffusion characteristics of the nuclear population of YFP-GR molecules (Fig. 1*D*). Given that the population of molecules is homogeneous, a single population of displacing molecules is determined with

$$P_{cum}\left(l,\Delta t\right)=1-ex[\left(-\frac{l^{2}}{MSD_{0}\left(\Delta t\right)}\right)$$

Here MSD_0_ is the mean square displacement of one population of molecules over the time lag. However, this one fraction model could not explain the experimental data (Fig. 1*D*). Therefore a second fraction was introduced and the equation reads as follows:

Where MSD_1_ and MSD_2_ denote the mean square displacement of the first (fast) and the second (slow) fractions respectively, and α is the fraction size of the first (fast) fraction. A two-population model fitted the experimental data with high accuracy (Fig. 1*D*). This analysis was repeated for each time lag and α, MSD_1_ and MSD_2_ were plotted against time (Δt). The displacements over time were best described using a free diffusion model in 2D (see for examples, Fig. 2*B*; Fig. S1*B*), from which the diffusion coefficients (D_fast_ and D_slow_) were calculated using the following equation:

$$MSD_{i}\left(\Delta t\right)=4\cdot D_{i}\cdot\Delta t$$

The fraction size α decreased slightly (on average -0.21 ± 0.04 %/ms) over increasing time lags in all groups (see Fig. S1*A*). Due to this effect, we always report the fraction distribution of the smallest time step (6.25 ms) as a representative of the overall fraction distribution.

**Monte Carlo quantification of FRAP curves.** The FRAP data was quantitatively analyzed by comparing the experimental data to curves generated using Monte Carlo modeling (3). The Monte Carlo computer simulations used to generate FRAP curves for the fit were based on a model that simulates diffusion of molecules in three dimensions and binding to immobile elements in an ellipsoidal volume. The laser bleach pulse was simulated based on experimentally derived three-dimensional laser intensity profiles, which were used to determine the probability for each molecule to get bleached considering their 3D position. The size of the ellipsoid model nucleus (x-, y- and z-diameters) was based on averages from experimental data. The simulation of the FRAP curve was then run using discrete time steps corresponding to the experimental scan interval of 100 ms. Diffusion was simulated at each new time step t + ∆t by deriving the new positions (x_t+∆t_, y_t+∆t_, z_t+∆t_) of all mobile molecules from their current positions (x_t_, y_t_, z_t_) by x_t+∆t_ = x_t_ + G(r_1_), y_t+∆t_= y_t_ + G(r_2_), and z_t+∆t_ = z_t_ + G(r_3_), where r_i_ is a random number (0 ≤ r_i_ ≤ 1) chosen from a uniform distribution, and G(r_i_) is the inverse of a cumulative Gaussian function with µ = 0 and σ^2^= 2D∆t, where D is the diffusion coefficient (obtained from SMM analysis). Immobilization was derived from simple binding kinetics described by:

$$\frac{k_{on}}{k_{off}}=\frac{F_{imm}}{F_{mob}}$$

where F_imm_ is the relative number of immobile molecules and F_mob_ = 1 - F_imm_. The probability for each particle to become immobilized (representing chromatin-binding) is defined as:

$$P_{imm}=k_{on}=\frac{F_{imm}}{\left(T_{imm}\cdot F_{mob}\right)}$$

where T_imm_ is the characteristic time spent in the immobile state. The probability to be released is given by:

$$P_{mob}=k_{off}=\frac{1}{T_{imm}}$$

As our model includes two bound fractions with different immobilization times, two immobilization/mobilization probabilities were evaluated for each unit time step. In all simulations, the size of the ellipsoid was based on the average size of measured nuclei, and the FRAP region used in the measurements determined the size of the simulated bleach region. The laser intensity profile using the simulation of the bleaching step was previously derived from confocal image stacks of chemically fixed nuclei containing GFP that were exposed to a stationary laser beam at various intensities and varying exposure times. The number of molecules in the simulations was 10^6^, which was empirically determined by producing curves that closely approximate the data with comparable fluctuations. The unit time step *∆t* corresponded to the experimental sample rate of 100 ms. When the simulation software was developed, the effect of laser scanning (especially during the bleachpulse) was investigated by running the simulation at very small time step of 1 microsecond, somewhat less than the pixel dwell time of the laser (pixel size 100 nm). In the applied strip-FRAP assay the laser scans ten lines of 512 pixels, and is only on - at high (bleaching) or low (monitoring) intensity- when scanning in the nucleus, which was included in the simulation. The simulation generated FRAP curves at this very short time step was not significantly different from the ones that are currently run routinely at 100 ms intervals.

**References**

1. Schaaf MJ & Cidlowski JA (2003) Molecular determinants of glucocorticoid receptor mobility in living cells: the importance of ligand affinity. *Mol Cell Biol* 23(6):1922-1934.

2. Semrau S & Schmidt T (2007) Particle image correlation spectroscopy (PICS): retrieving nanometer-scale correlations from high-density single-molecule position data. *Biophys J* 92(2):613-621.

3. van Royen ME*, et al.* (2009) Fluorescence recovery after photobleaching (FRAP) to study nuclear protein dynamics in living cells. *Methods Mol Biol* 464:363-385.
